# 634. Association of pre-transplant CD4 count on post-renal transplant infectious outcomes among persons with HIV

**DOI:** 10.1093/ofid/ofae631.199

**Published:** 2025-01-29

**Authors:** Rebecca H Burns, Christin Rogers Marks, Sara Booth, Christine Durand, Jonathan Hand, Maheen Abidi, Maricar F Malinis, Brittany Barnaba, Martha Pavlakis, Douglas Krakower, Carolyn D Alonso, Audrey Le Mahajan

**Affiliations:** Warren Alpert Medical School of Brown University, Boston, MA; Beth Israel Deaconess Medical Center, Norton, Massachusetts; Beth Israel Deaconess Medical Center, Norton, Massachusetts; Johns Hopkins, Baltimore, MD; Ochsner Health, New Orleans, LA; University of Colorado Anschutz Medical Campus, Denver, CO; Vanderbilt University Medical Center, TN; Johns Hopkins University School of Medicine, Baltimore, Maryland; Beth Israel Deaconess Medical Center, Harvard Medical School, Boston, MA; Harvard Medical School, Boston, MA; Beth Israel Deaconess Medical Center, Norton, Massachusetts; Beth Israel Deaconess Medical Center, Norton, Massachusetts

## Abstract

**Background:**

Many persons with HIV (PWH) are excluded as kidney transplants (KT) recipients due to guidelines recommending pre-transplant CD4 counts > 200 cells/mL. Historically, this threshold has been suggested due to concern for increased rates of infection among patients with lower CD4 counts, however, it has not been rigorously studied and may be unnecessarily stringent leading to exclusion of otherwise acceptable organ candidates.

**Figure:**
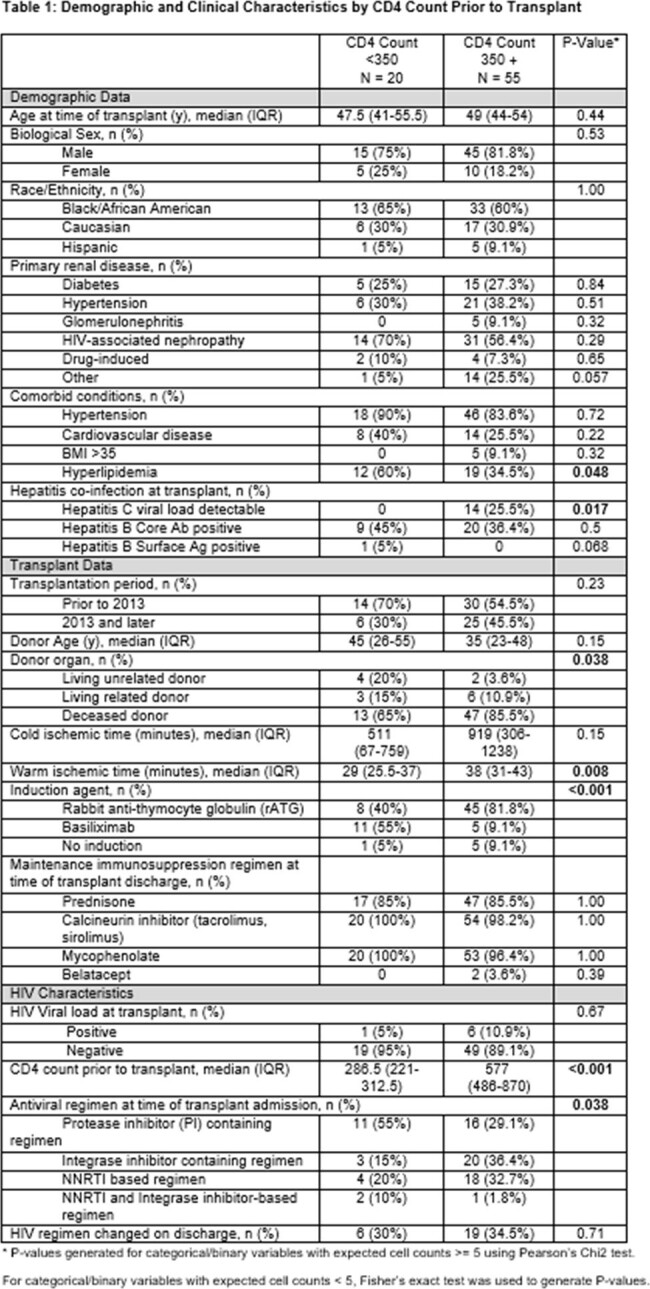

**Methods:**

We performed a multicenter, retrospective cohort study of post-transplant infectious outcomes in PWH undergoing KT from 2004 - 2019. We defined our exposure of interest as a pre-transplant CD4 count < 350 cells/µL based on prior literature. Our primary outcome was presence of infection (outpatient or inpatient) at 1 year post-transplant. Secondary outcomes included time-to-first infection and infections requiring hospitalization. Multivariable logistic regression analysis was performed for the primary outcome, adjusting for confounding variables.

**Figure:**
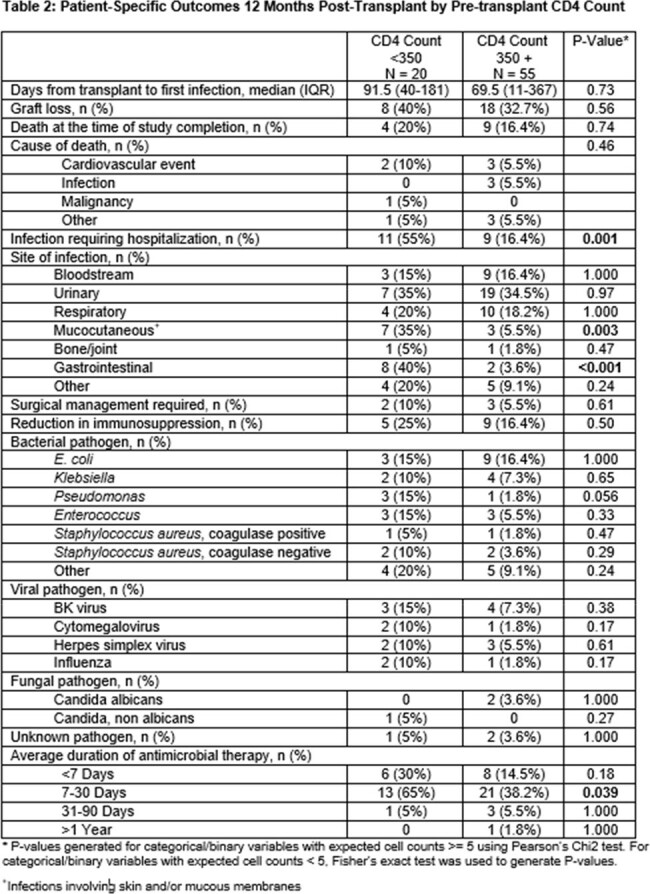

**Results:**

Of the 75 patients included, 20 (27%) had pre-transplant CD4 counts < 350. There were no differences in baseline demographics (Table 1). The CD4 < 350 group was more likely to have hyperlipidemia (p 0.048), receive a protease-inhibitor based regimen (p 0.042) and to undergo living-donor organ donation (p 0.05). The CD4 ≥ 350 group was more likely to have Hepatitis C (p 0.017), receive anti-thymocyte globulin induction and experience longer warm ischemic times (p 0.008). There were no significant differences in time-to-first infection, graft loss or death (Table 2). Patients with CD4 < 350 were more likely to have mucocutaneous (p 0.003) and gastrointestinal infections (p < 0.001) and be hospitalized for infection (p 0.028). After adjusting for confounders, CD4 < 350 was not associated with an increased risk of infection in the first year post-KT (aOR 5.28; p 0.13; 95% CI: 0.61, 45.6; Table 3).

**Figure:**
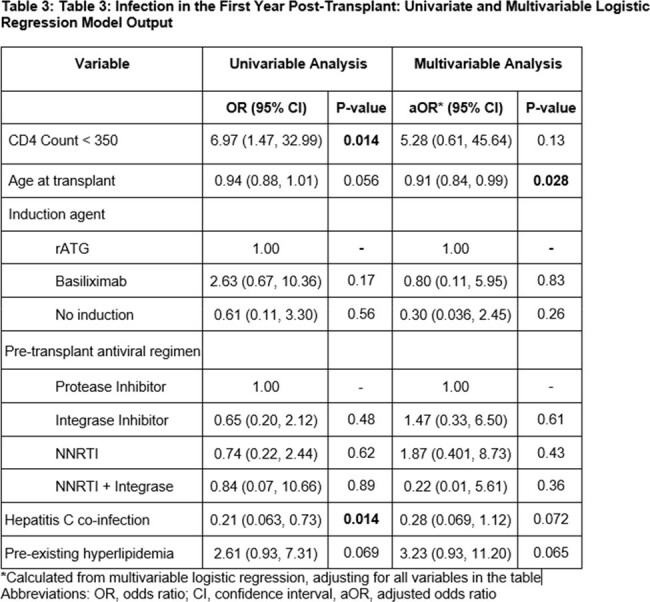

**Conclusion:**

Lower pre-transplant CD4 count was not associated with an increased risk of post-transplant infection, graft-loss or death, although patients with CD4 < 350 were more likely to be hospitalized. Future studies are needed to determine if organ transplant CD4 threshold levels should be recalibrated in PWH.

**Disclosures:**

**Christin Rogers Marks, PharmD**, Novo Nordisk: Current employee **Christine Durand, MD**, Gilead: Grant/Research Support|Gilead: Honoraria **Jonathan Hand, MD**, AstraZeneca: Grant/Research Support|Janssen: Grant/Research Support|Pfizer: Advisor/Consultant|Pfizer: Grant/Research Support|Scynexis: Grant/Research Support **Maricar F. Malinis, MD**, Moderna ( concluded): Advisor/Consultant|Paratek Pharmaceuticals, Inc.: Honoraria **Martha Pavlakis, MD**, Merck: Advisor/Consultant|Vertex: Advisor/Consultant **Douglas Krakower, MD**, FluidForm Bio: Stocks/Bonds (Private Company)|Gilead: Grant/Research Support|Matchbox Health: Stocks/Bonds (Private Company)|Medscape: Funding to develop educational content|Merck: Grant/Research Support|PrEP4All: Travel to meeting on PrEP acccess **Carolyn D. Alonso, MD**, AiCuris: Advisor/Consultant|Cidara: Advisor/Consultant|Ferring: Travel support to present data at meeting|Merck: Advisor/Consultant|Pfizer: Advisor/Consultant **Audrey Le Mahajan, MD**, AstraZeneca: Grant/Research Support

